# The Impact of Preoperative Biliary Drainage on Postoperative Outcomes Following Pancreaticoduodenectomy: A Systematic Review

**DOI:** 10.7759/cureus.95569

**Published:** 2025-10-28

**Authors:** Mohey Aldien Ahmed Elamin Elnour, Mohamed Abdelmagid Eltahir Hamza, Eiman Mohamed Abdelrazik, Manal Hashim Saeid Othman, Khaled Abdulbasit Fadlallah Mohammed, Seedahmed Mohamed Seedahmed Kunna, Mawahib Sabir Abdalla Abdalrasoul

**Affiliations:** 1 Trauma and Orthopaedics, Royal Derby Hospital, Derby, GBR; 2 Surgery, King Salman Armed Forces Hospital, Tabuk, SAU; 3 General Surgery, Our Lady's Hospital, Navan, IRL; 4 General Surgery, King Fahad Hospital, Madina, SAU; 5 General Surgery, University Hospitals Coventry and Warwickshire NHS Trust, Coventry, GBR; 6 General Surgery, University Hospital Kerry, Tralee, IRL; 7 General Surgery, Al-Fashir Teaching Hospital, Al-Fashir, SDN

**Keywords:** obstructive jaundice, pancreaticoduodenectomy, postoperative outcomes, preoperative biliary drainage, surgical site infection, systematic review, whipple procedure

## Abstract

Pancreaticoduodenectomy (PD) is the standard procedure for periampullary and pancreatic head malignancies, often performed in the context of obstructive jaundice. Preoperative biliary drainage (PBD) is used to alleviate jaundice, but its impact on postoperative outcomes remains controversial due to conflicting evidence regarding its risks and benefits. This systematic review aims to synthesize the most recent evidence on the impact of PBD on postoperative outcomes following PD.

A systematic literature search was conducted across five electronic databases (PubMed, Scopus, Web of Science, Embase, CINAHL) for studies published between 2020 and 2025. Twelve studies, comprising retrospective cohort analyses, were included after a rigorous screening process. Data on study characteristics, patient populations, and postoperative outcomes were extracted. The risk of bias was assessed using the ROBINS-I tool, and a qualitative synthesis was performed due to the heterogeneity of the included studies. The evidence indicates a dualistic impact of PBD. It is consistently associated with a higher risk of infectious complications, including surgical site and intra-abdominal infections. However, the preoperative bilirubin level is a critical effect modifier; in patients with severe hyperbilirubinemia, PBD appears to mitigate the high risks of overall morbidity and mortality associated with profound jaundice. The effect of PBD on specific complications like postoperative pancreatic fistula and delayed gastric emptying was ambiguous. The timing of PBD also influences outcomes, with shorter intervals potentially reducing infectious risks. The overall methodological quality of the included studies was predominantly low to moderate risk of bias. The decision to implement PBD should not be universal but individualized. While PBD introduces a significant risk of infectious morbidity, it may be a crucial risk-mitigation strategy in severely jaundiced patients. The procedure's benefits are likely contingent on specific bilirubin thresholds and careful consideration of drainage timing. Future research should focus on validating these thresholds and optimizing perioperative protocols.

## Introduction and background

Pancreaticoduodenectomy (PD), commonly known as the Whipple procedure, remains the cornerstone surgical intervention for periampullary and pancreatic head malignancies [1]. Despite advances in surgical techniques, anesthesia, and perioperative care, PD continues to be associated with substantial morbidity and mortality [2]. One of the significant challenges in the perioperative management of these patients is obstructive jaundice, which is commonly encountered due to malignant biliary obstruction [3]. Prolonged jaundice can result in impaired liver function, coagulopathy, malnutrition, and increased susceptibility to infections, potentially complicating postoperative recovery [4].

Preoperative biliary drainage (PBD) has been proposed as a strategy to alleviate the effects of obstructive jaundice before surgery [5]. By restoring bile flow, PBD aims to improve hepatic function, correct coagulation abnormalities, and potentially reduce postoperative complications. However, PBD is an invasive procedure and carries its own risks, including procedure-related infections, stent occlusion, pancreatitis, and bleeding [6]. These complications may negate the theoretical benefits of preoperative drainage and could influence overall postoperative outcomes.

The role of PBD in patients undergoing PD has been the subject of ongoing debate. While some studies suggest that PBD reduces postoperative morbidity and improves liver function, others report increased rates of infectious complications and longer hospital stays [7]. Given the heterogeneity in study designs, patient populations, drainage techniques, and timing of surgery, the impact of PBD on postoperative outcomes remains unclear.

This systematic review aims to synthesize the most recent evidence to evaluate the effect of PBD on postoperative outcomes following PD. By examining both the benefits and potential harms associated with PBD, this review seeks to provide clinicians with a comprehensive understanding of its role in perioperative management and inform evidence-based decision-making in patients with malignant biliary obstruction.

## Review

Methods

Eligibility Criteria

This systematic review included studies evaluating the impact of PBD on postoperative outcomes following PD. Only studies published in the last five years (2020-2025) were considered to ensure that the review reflects the most recent evidence and contemporary surgical and perioperative practices. Eligible studies included randomized controlled trials (RCTs), prospective and retrospective cohort studies, and observational studies that directly compared outcomes between patients undergoing PBD and those proceeding to surgery without PBD (No-PBD group). Studies focusing solely on pediatric populations, animal models, or non-relevant surgical procedures were excluded.

Information Sources

A comprehensive literature search was conducted across multiple databases, including PubMed/MEDLINE, Embase, Web of Science, Scopus, and CINAHL. In addition to database searches, studies were identified through citation searching of relevant articles to capture potentially eligible studies not indexed in these databases. The search strategy was designed to maximize sensitivity while focusing on relevant studies published within the defined timeframe.

Search Strategy

The search strategy included keywords and MeSH terms related to “preoperative biliary drainage,” “pancreaticoduodenectomy,” “Whipple procedure,” “postoperative outcomes,” and “complications.” Boolean operators, truncation, and database-specific filters were applied to refine the search results. The full search strategies for all databases are available in the supplementary materials.

Study Selection

All retrieved studies were imported into EndNote, where duplicates were removed. Two independent reviewers screened titles and abstracts for eligibility, followed by full-text reviews to determine final inclusion. Discrepancies were resolved through discussion, and a third reviewer was consulted when consensus could not be reached.

Data Collection Process

Data were extracted independently by two reviewers using a standardized extraction form. Extracted information included study characteristics (country, design, sample size), patient demographics, indication for PBD, type of intervention, comparator, timing to surgery, and postoperative outcomes including overall morbidity, infectious complications, pancreatic fistula, delayed gastric emptying, mortality, and length of hospital stay.

Data Synthesis

Due to considerable heterogeneity among studies in terms of study design, patient populations, indications for PBD, type of drainage, timing to surgery, and reporting of outcomes, a meta-analysis was not performed. Instead, a qualitative synthesis of the evidence was conducted to summarize trends and highlight differences in postoperative outcomes between PBD and No-PBD groups. This approach allowed for a more nuanced interpretation of the available evidence without introducing potential bias from pooling heterogeneous data.

Risk of Bias Assessment

The risk of bias for included observational studies was assessed using the ROBINS-I tool [8], which evaluates bias across domains including confounding, selection of participants, classification of interventions, deviations from intended interventions, missing data, measurement of outcomes, and selection of reported results. The assessment was performed independently by two reviewers, and any disagreements were resolved through discussion or consultation with a third reviewer, following the recommendations of the Cochrane Handbook for Systematic Reviews of Interventions. Studies were then categorized as low, moderate, serious, or critical risk of bias based on these criteria.

Results

Study Selection Process

**Figure 1:**
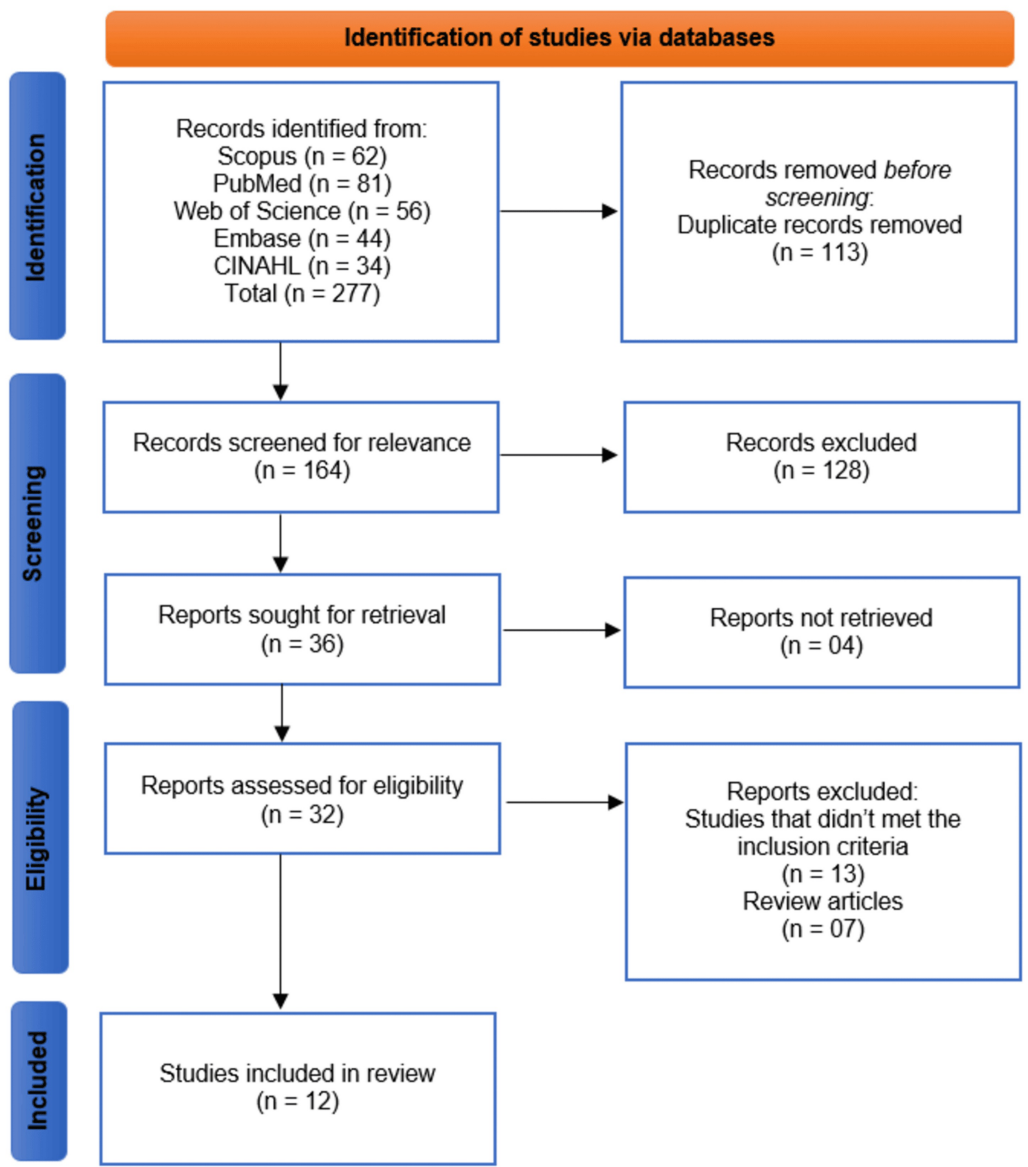
Illustration of the Selection Process of the Studies on a PRISMA Flowchart PRISMA, Preferred Reporting Items for Systematic Reviews and Meta-Analyses

The systematic search across five databases (Scopus, PubMed, Web of Science, Embase, and CINAHL) initially identified 277 records. After the removal of 113 duplicate records, a total of 164 unique publications were screened based on their titles and abstracts. This screening process led to the exclusion of 128 records that were not relevant to the review's objective. The full texts of the remaining 36 reports were sought for retrieval, of which 4 could not be accessed. Consequently, 32 full-text articles were assessed for eligibility. Upon detailed evaluation, 20 articles were excluded, with the primary reasons being that they did not meet the predefined inclusion criteria (n=13) or were review articles (n=07). Ultimately, 12 studies were selected for inclusion in the final systematic review [9-20]. The study selection process is detailed in the Preferred Reporting Items for Systematic Reviews and Meta-Analyses (PRISMA) flow diagram (Figure 1).

Characteristics of Included Studies

A total of 12 studies [9-20] were included in this systematic review, comprising retrospective cohort analyses, with two studies utilizing data from the American College of Surgeons National Surgical Quality Improvement Program (ACS-NSQIP) [13,17]. The publication years of the included studies ranged from 2020 to 2025, reflecting a contemporary analysis of the topic. The studies were conducted across a diverse range of geographical regions, including China [10-14,16], the United States [13,17], Israel [9], Denmark [18], Pakistan [19], and Spain [20]. The sample sizes varied considerably, from smaller cohorts of 123 patients [20] to large, multi-institutional analyses of over 8,000 patients [13]. The primary population across all studies was patients undergoing PD, most commonly for periampullary or pancreatic malignancy in the context of obstructive jaundice or hyperbilirubinemia. The intervention of interest was PBD, achieved via endoscopic (ERCP) or percutaneous (PTBD) methods, which was compared to a strategy of upfront surgery without drainage (No-PBD). The timing from PBD to surgery was investigated as a modifying factor in several studies [11,12,16,19]. The characteristics of the included studies are summarized in Table 1.

**Table 1:** Characteristics of Included Studies PBD, Preoperative Biliary Drainage; No-PBD, No Preoperative Biliary Drainage; PD, Pancreaticoduodenectomy; ERCP, Endoscopic Retrograde Cholangiopancreatography; PTBD, Percutaneous Transhepatic Biliary Drainage; LPD, Laparoscopic Pancreaticoduodenectomy; PSM, Propensity Score Matching; NR, Not Reported; SSI, Surgical Site Infection; ARDS, Acute Respiratory Distress Syndrome; HAIs, Healthcare-Associated Infections; ICU, Intensive Care Unit; LOS, Length of Stay; PAC, Pancreatic Adenocarcinoma; DGE, Delayed Gastric Emptying; PPH, Post-pancreatectomy Hemorrhage; POPF, Postoperative Pancreatic Fistula.

Author (Year)	Country/Region	Study Design	Sample Size (n)	Population Characteristics	Indication for PBD	Intervention (PBD Group)	Comparator (No-PBD Group)	Timing to Surgery	Primary Outcomes Reported	Secondary Outcomes Reported	Main Findings
Kanani et al., [9] (2025)	Israel	Retrospective cohort	665	PD patients, stratified by bilirubin >14.6 or <14.6 mg/dL	Severe hyperbilirubinemia (>14.6 mg/dL)	PBD (stratified by bilirubin: >14.6 mg/dL, n=113; <14.6 mg/dL, n=140)	No PBD (stratified by bilirubin: >14.6 mg/dL, n=83; <14.6 mg/dL, n=312)	After PBD (not specified)	90-day mortality, morbidity	SSI, ARDS, reoperation	No-PBD with high bilirubin had ↑mortality & morbidity; PBD ↑SSI; high bilirubin independently predicted mortality
Li et al., [10] (2025)	China (Chongqing)	Retrospective	326	PD patients (Oct 2018–Jul 2023)	High bilirubin (≥171 μmol/L)	PBD	No PBD	NR	PD complications	Bilirubin-complication relationship	High bilirubin ↑ complications; PBD ↓ risk if bilirubin ≥171 μmol/L
Yu et al., [11] (2024)	China	Retrospective cohort	2842	PD patients	Likely biliary obstruction	PBD	No PBD	Interval analyzed (≤2 vs ≥4 wks)	Post-op HAIs	ICU transfer, LOS	PBD ↑HAIs in robotic PD, ↑ICU & LOS; timing modifies risk
Zhu et al., [12] (2023)	China	Retrospective cohort	148 (PBD: 98, No-PBD: 50)	Obstructive jaundice, PAC	Obstructive jaundice	PBD (short drain-to-surgery interval: ≤2 weeks, n=49; long drain-to-surgery interval: >2 weeks, n=49)	No PBD	Mean 13 days	Postop intra-abdominal infection	Ascites culture, bile/peritoneal pathogens	PBD ↑ infection; short-term safe; long-term ↑ positive cultures; bile pathogens linked to infection
Werba et al., [13] (2022)	USA (ACS-NSQIP)	Retrospective, propensity-matched	8,970 (4,473/829 obstructed; 711/2,957 non-jaundiced)	PD for periampullary malignancy (2014-2018)	Obstructive jaundice	Preoperative stent	No drainage	Prior to PD	30-day mortality, major morbidity	Superficial/deep/organ-space SSI, wound dehiscence	No increase in mortality/morbidity; ↑ superficial SSI; ↑ wound issues in non-obstructed
Wang et al., [14] (2021)	China (Single hospital)	Retrospective cohort, PSM	172 (48 each, matched)	Malignant obstructive jaundice	Malignant obstructive jaundice	PTBD + LPD	LPD alone	NR	Complications, hemorrhage, DGE, operative time, blood loss	Catheter complications, oncologic outcomes	PTBD reduced overall complications & hemorrhage; other outcomes NS after matching
Shen et al., [15] (2020)	Not specified	Retrospective cohort, PSM	200	Severe obstructive jaundice, TBil >250 μmol/L	Obstructive jaundice	PBD before PD	Direct surgery	Jan 2012–Dec 2017	Overall postoperative complications	Operative time, transfusion, PPH, POPF	PBD reduced postoperative complications, PPH, and POPF
Ray et al., [16] (2021)	China	Retrospective	404 (PBD: 175 / No-PBD: 229)	Median age 50; 63% male	High bilirubin / clinical need	Preop biliary drainage	No drainage	Median 59 days	Overall complications, SSI	POPF, DGE, collections, hemorrhage, mortality, hospital stay	PBD ↑ overall complications & SSI; no effect on major complications or mortality; >6 weeks interval safe; bilirubin alone not beneficial
Garcia-Ochoa et al., [17] (2021)	USA (ACS-NSQIP)	Retrospective cohort, propensity-matched	6073 total; PBD: 3467, No-PBD: 952 (matched 4419)	Pancreatic cancer with obstructive jaundice	Obstructive jaundice	Pre-op ERCP stent	No stent	Before surgery	30-day post-op complications	Perioperative complications	No increase in post-op complications; stenting safe
Farooqui et al., [18] (2022)	Denmark	Retrospective cohort	722 (389/333)	PD patients	Obstructive jaundice	ERC/PTC drainage	No drainage	NR	Post-op complications, mortality	Drainage complications	PBD safe, fewer complications, lower mortality
Bhatti et al., [19] (2021)	Pakistan	Retrospective cohort	66 / 67	PD for obstructive jaundice	Obstructive jaundice	Preoperative biliary stent	Upfront surgery	4–6 weeks (high-risk)	30- & 90-day mortality, Pancreatic fistula	Wound infection, Readmission	PBS ↑ wound infection; no difference in mortality/fistula
Bademci et al., [20] (2022)	Spain	Retrospective cohort	123 (PBD:48, No-PBD:75)	Periampullary tumour; subgroup: pancreatic head	Obstructive jaundice	ERCP stent (Metal:31, Plastic:17)	No PBD	NR	Operative & postop complications	Wound infection; stent type comparison	Similar outcomes except higher wound infection in PBD group; no stent type difference

Impact on Overall Postoperative Morbidity

The impact of PBD on overall postoperative morbidity was a key outcome with conflicting results across the studies. Several studies reported a beneficial or neutral effect. Shen et al. [15] found that PBD significantly decreased overall postoperative complications in patients with severe obstructive jaundice (total bilirubin >250 μmol/L). Similarly, Wang et al. [14] reported that PTBD reduced overall complications in patients with malignant obstructive jaundice undergoing laparoscopic PD. In a large propensity-matched analysis, Garcia-Ochoa et al. [17] concluded that preoperative stenting did not increase postoperative complications, and Farooqui et al. [18] observed fewer complications in the PBD group.

Conversely, other studies reported increased morbidity associated with PBD. Ray et al. [16] found that PBD was associated with a significantly higher rate of overall complications (57.7% vs. not reported for No-PBD, but context suggests higher). Kanani et al. [9] reported that while high bilirubin was an independent predictor of poor outcomes, PBD itself was associated with increased morbidity in certain subgroups. Notably, Li et al. [10] highlighted that the relationship was complex and dependent on bilirubin levels, with PBD reducing complications only when the preoperative bilirubin was ≥171 μmol/L, but not independently affecting the overall complication rate.

Impact on Infectious Complications and Surgical Site Infections

A consistently reported finding across multiple studies was an increased risk of infectious complications, particularly surgical site infections (SSIs), in patients who underwent PBD. Werba et al. [13] found that while PBD did not increase mortality or major morbidity, it was associated with a higher rate of superficial SSI and wound dehiscence. This was corroborated by Bhatti et al. [19], who reported a higher rate of wound infection in the PBD group (22.7% vs. 7.4%), and Bademci et al. [20], who also noted a higher wound infection rate in the PBD cohort. Zhu et al. [12] specifically reported a significantly higher rate of postoperative intra-abdominal infection in the PBD group, which was linked to the presence of pathogens in the bile. Furthermore, Yu et al. [11] found that PBD was associated with an increase in postoperative healthcare-associated infections (HAIs), particularly in robotic PD procedures. Kanani et al. [9] also reported a notably higher rate of infectious complications in the PBD group (21.2-26.4%) compared to the No-PBD group (9.6%).

Impact on Specific Postoperative Complications

The effect of PBD on other specific postoperative complications was also investigated. Regarding postoperative pancreatic fistula (POPF), the evidence was mixed. Shen et al. [15] reported a lower rate of clinically relevant POPF (Grades B & C) in the PBD group, suggesting a protective effect. In contrast, Bhatti et al. [19] found no significant difference in POPF rates between groups, and Ray et al. [16] did not report a significant effect of PBD on major complications like POPF.

For delayed gastric emptying (DGE), Wang et al. [14] observed a lower rate in the PTBD group compared to the No-PBD group (4.17% vs. 12.50%), while Ray et al. [16] reported a DGE rate of 20% without a direct comparison to a No-PBD group. Post-pancreatectomy hemorrhage (PPH) was significantly reduced in the PBD groups in studies by Shen et al. [15] and Wang et al. [14].

Impact on Postoperative Mortality

The majority of studies indicated that PBD did not significantly increase postoperative mortality. Werba et al. [13] and Garcia-Ochoa et al. [17] both reported no increase in 30-day mortality. Notably, Farooqui et al. [18] found that PBD was associated with significantly lower 30-day and 90-day mortality. However, the critical factor appeared to be the underlying bilirubin level. Kanani et al. [9] demonstrated that patients with high bilirubin levels (>14.6 mg/dL) who did not undergo PBD had substantially higher mortality (13.3%) compared to all other groups (2.9-5.7%), indicating that high bilirubin itself is a primary risk factor and that the decision for PBD must be weighed against this background risk. Bhatti et al. [19] also found no significant difference in 30-day or 90-day mortality between groups. A summary of these postoperative outcomes is presented in Table 2.

**Table 2:** Summary of Postoperative Outcomes Across Studies PBD, Preoperative Biliary Drainage; No-PBD, No Preoperative Biliary Drainage; NR, Not Reported; SSI, Surgical Site Infection; HAIs, Healthcare-Associated Infections; POPF, Postoperative Pancreatic Fistula; DGE, Delayed Gastric Emptying; DS, Direct Surgery.

Author (Year)	Sample Size (PBD / No-PBD)	Overall Morbidity (%)	Infectious Complications (%)	Postoperative Pancreatic Fistula (%)	Delayed Gastric Emptying (%)	Postoperative Mortality (%)
Kanani et al., [9] (2025)	PBD (n = 253) / No-PBD (n = 395)	12.8–20.7% (PBD) vs 29.0% (No-PBD, high bilirubin)	21.2–26.4% (PBD) vs 9.6% (No-PBD)	NR	NR	2.9–5.7% (PBD & No-PBD low bilirubin) vs 13.3% (No-PBD high bilirubin)
Li et al., [10] (2025)	326 total	Higher preoperative bilirubin associated with increased morbidity; PBD did not independently affect complications, but reduced complications when bilirubin ≥ 171 μmol/L	NR	NR	NR	NR
Yu et al., [11] (2024)	2842	NR	8.7 (HAIs)	NR	NR	NR
Zhu et al., [12] (2023)	98/50	NR	Higher in PBD vs No-PBD (P=0.026)	NR	NR	NR
Werba et al., [13] (2022)	4,473/829 (obstructive) & 711/2,957 (non-jaundiced)	19.8–20	Superficial SSI: 6.8–9.2; wound dehiscence: 0.5–1.5	NR	NR	2.2–2.4
Wang et al., [14] (2021)	48/48 (propensity-matched)	22.92 / 39.58	NR	NR	4.17 / 12.50	NR
Shen et al., [15] (2020)	200 (exact PBD / DS split not specified)	Higher in DS group; 59.5% overall	NR	Higher in DS group (Grades B & C POPF, p = 0.045)	NR	NR
Ray et al., [16] (2021)	175/229	57.7	28.7	14.1	20	4.2
Garcia-Ochoa et al., [17] (2021)	3467/952	55 / 53	NR	NR	NR	NR
Farooqui et al., [18] (2022)	389/333	23 (PBD) / Higher in No-PBD	NR	NR	NR	Lower in PBD (30-day & 90-day mortality significant)
Bhatti et al., [19] (2021)	66/67	NR	22.7 / 7.4	7.5 / 4.4	NR	30-day: 3 / 2.990-day: 7.5 / 4.4
Bademci et al., [20] (2022)	48/75	NR	Wound infection ↑ in PBD	NR	NR	NR

The Role of Preoperative Bilirubin Level and Timing of Drainage

The preoperative bilirubin level was identified as a crucial effect modifier. Kanani et al. [9] and Li et al. [10] both emphasized that severe hyperbilirubinemia was an independent predictor of increased mortality and morbidity. PBD appeared to be most beneficial, or at least warranted, in this high-bilirubin subgroup [9, 10, 15]. Conversely, for patients with lower bilirubin levels, PBD seemed to offer no benefit and potentially increased the risk of infectious complications [9, 16].

The timing between PBD and surgery was another significant factor. Zhu et al. [12] found that while short-term drainage (≤2 weeks) was relatively safe, long-term drainage (>2 weeks) led to a higher rate of positive bile cultures and infections. Yu et al. [11] also reported that the timing interval modified the risk of HAIs, and Ray et al. [16] suggested that an interval of more than six weeks between PBD and surgery might be safer. Bhatti et al. [19] specifically investigated timing but focused on high-risk patients drained 4-6 weeks preoperatively.

Risk of Bias Assessment

The methodological quality of the included studies was assessed using the ROBINS-I tool, with the results summarized in Table 3. The overall risk of bias varied across the twelve studies. The majority of studies were judged to have a low risk of bias [10-13,15-17,19], indicating that their methodology was robust and their findings are likely reliable. Three studies were assessed as having a moderate risk of bias [9,14,20], primarily due to potential residual confounding despite the use of statistical adjustments like propensity score matching. One study, Farooqui et al. [18], was rated as having a serious risk of bias, suggesting important limitations, likely in controlling for key confounding variables, which necessitates a more cautious interpretation of its findings. For all studies, the risk of bias was low across other domains, including selection of participants, classification of interventions, and measurement of outcomes, indicating strong methodological conduct in these areas.

**Table 3:** Risk of Bias Assessment Using the ROBINS-I Tool

Author (Year)	D1: Bias Due to Confounding	D2: Bias in the Selection of Participants	D3: Bias in the Classification of Interventions	D4: Bias Due to Deviations From Intended Interventions	D5: Bias Due to Missing Data	D6: Bias in the Measurement of Outcomes	D7: Bias in the Selection of the Reported Result	Overall Risk of Bias
Kanani et al., [9] (2025)	Moderate	Low	Low	Low	Low	Low	Low	Moderate
Li et al., [10] (2025)	Low	Low	Low	Low	Low	Low	Low	Low
Yu et al., [11] (2024)	Low	Low	Low	Low	Low	Low	Low	Low
Zhu et al., [12] (2023)	Low	Low	Low	Low	Low	Low	Low	Low
Werba et al., [13] (2022)	Low	Low	Low	Low	Low	Low	Low	Low
Wang et al., [14] (2021)	Moderate	Low	Low	Low	Low	Low	Low	Moderate
Shen et al., [15] (2020)	Low	Low	Low	Low	Low	Low	Low	Low
Ray et al., [16] (2021)	Low	Low	Low	Low	Low	Low	Low	Low
Garcia-Ochoa et al., [17] (2021)	Low	Low	Low	Low	Low	Low	Low	Low
Farooqui et al., [18] (2022)	Serious	Low	Low	Low	Low	Low	Low	Serious
Bhatti et al., [19] (2021)	Low	Low	Low	Low	Low	Low	Low	Low
Bademci et al., [20] (2022)	Moderate	Low	Low	Low	Low	Low	Low	Moderate

Discussion

This systematic review, encompassing 12 contemporary studies, provides a comprehensive and nuanced synthesis of the current evidence regarding the impact of PBD on postoperative outcomes following PD. The central finding of this analysis is that the relationship between PBD and patient outcomes is not monolithic but is profoundly influenced by two critical, interdependent factors: the patient's baseline level of hyperbilirubinemia and the inherent infectious risks associated with the drainage procedure itself. Our results paint a complex picture, suggesting that while PBD can mitigate the dangers of profound jaundice, it concurrently introduces a significant risk of infectious morbidity, thereby creating a delicate therapeutic balance for the clinician.

The most striking and consistent finding across the included studies is the heightened risk of infectious complications associated with PBD. As demonstrated by Werba et al. [13], Bhatti et al. [19], and Bademci et al. [20], patients undergoing PBD faced a significantly increased incidence of surgical site infections and wound complications. This is further substantiated by Zhu et al. [12], who linked this risk to bacterial colonization, finding that long-term drainage was associated with higher rates of positive bile cultures and subsequent intra-abdominal infections. The biological plausibility for this is strong; endoscopic or percutaneous intervention inherently breaches the sterile biliary tree, exposing it to the contaminating environment of the gastrointestinal tract or skin, thereby seeding a potential source for postoperative infection [21]. This finding aligns with the established literature. A large meta-analysis by Scheufele et al. [22] concluded that PBD significantly increased the rate of overall complications, primarily driven by infectious sequelae. Similarly, a study by Fu et al. [23] found that PBD was an independent risk factor for postoperative infectious complications, reinforcing the notion that the procedure itself, while relieving obstruction, creates a new set of challenges.

However, interpreting this increased infectious risk as a blanket contraindication for PBD would be an oversimplification. The data compellingly illustrate that the preoperative bilirubin level is a paramount effect modifier. The works of Kanani et al. [9] and Li et al. [10] are pivotal in this regard, demonstrating that severe hyperbilirubinemia is an independent and powerful predictor of both increased mortality and overall morbidity. Kanani et al. [9] showed that patients with high bilirubin levels who did not undergo PBD suffered a mortality rate of 13.3%, starkly higher than the 2.9-5.7% observed in other groups. This suggests that the risk of forgoing drainage in a severely jaundiced patient may outweigh the infectious risk posed by the procedure. This is corroborated by Li et al. [10], who found that PBD reduced complications specifically in the subgroup with bilirubin ≥171 μmol/L. The protective mechanism is likely multifactorial, involving the amelioration of cholestasis-induced immune dysfunction, renal impairment, and impaired wound healing associated with profound jaundice [24]. Our findings thus support a risk-stratified approach, echoing the conclusions of a landmark randomized controlled trial by van der Gaag et al. [25], which, while cautioning against routine PBD, acknowledged its necessity in selected patients with deep jaundice or those facing delayed surgery. Our review refines this further, suggesting that the bilirubin thresholds identified by Kanani et al. [9] (>14.6 mg/dL) and Li et al. [10] (≥171 μmol/L) provide practical, evidence-based benchmarks for this critical decision.

Beyond overall morbidity and infectious complications, the impact of PBD on specific surgical endpoints remains ambiguous. The evidence for POPF is particularly conflicted. Shen et al. [15] reported a lower rate of clinically relevant POPF in the PBD group, potentially due to improved nutritional status and decreased tissue friability in decompressed patients. In contrast, Bhatti et al. [19] found no difference, and Ray et al. [16] did not identify a significant effect. This discrepancy may be explained by variations in surgical technique, fistula definition, and, crucially, the baseline pancreatic texture and duct size of the study populations, factors known to be the most potent predictors of POPF [26]. For delayed gastric emptying, the data from Wang et al. [14] suggested a benefit for PBD, whereas Ray et al. [16] reported a high rate without a clear comparative advantage. The reduction in post-pancreatectomy hemorrhage observed by Shen et al. [15] and Wang et al. [14] is a notable finding, possibly related to the correction of the coagulopathy often present in jaundiced patients. This aligns with the pathophysiology of obstructive jaundice, where malabsorption of vitamin K can lead to a correctable coagulopathy, and PBD allows for its normalization prior to major surgery [27].

The timing of PBD in relation to surgery emerged as another critical variable influencing outcomes. The analysis by Zhu et al. [12] provides a compelling argument against prolonged drainage, demonstrating that intervals exceeding two weeks promote bacterial colonization and increase infectious risk. This is a crucial insight for clinical scheduling, advocating for a strategy of "shortest feasible interval" once drainage is deemed necessary. Yu et al. [11] and Ray et al. [16] also highlighted that the timing window modifies risk, with the latter suggesting an interval of over six weeks might be safer, a finding that appears to contradict Zhu et al. [12]. This discrepancy may be related to different patient populations, drainage techniques, or perioperative management protocols, and it underscores the need for further prospective investigation to define the optimal timing. The finding from Bhatti et al. [19], focusing on a 4-6 week window for high-risk patients, adds another layer, suggesting that in complex cases, a longer, planned period of optimization may be beneficial, potentially allowing for nutritional support and treatment of comorbidities. This concept of "pre-habilitation" is gaining traction in complex pancreatic surgery [28].

When considering mortality, the consensus from this review is reassuring; PBD, when appropriately applied, does not appear to increase postoperative mortality. The large, propensity-matched studies by Werba et al. [13] and Garcia-Ochoa et al. [17] found no increase in 30-day mortality. Importantly, Farooqui et al. [18] even reported lower mortality in the PBD group, though this study's serious risk of bias necessitates cautious interpretation. The critical insight, again provided by Kanani et al. [9], is that mortality is more closely tied to the underlying bilirubin level than to the drainage procedure per se. This nuanced view is supported by the study by Coates et al. [29], which concluded that the negative impact of PBD observed in earlier studies was likely confounded by indication, with sicker, more jaundiced patients being selected for drainage. Our findings, particularly from the higher-quality propensity-matched studies [13,17], suggest that in well-matched cohorts, PBD itself is not a direct cause of death, but rather a modulator of specific morbidities.

The methodological assessment of the included evidence reveals a landscape dominated by retrospective studies, a inherent limitation in this field. However, the use of propensity score matching in several studies [9,13-15,17] strengthens their validity by mitigating the profound confounding by indication-whereby patients selected for PBD are inherently higher risk. The overall low to moderate risk of bias in the majority of studies [9-20] lends confidence to the synthesized findings. The single study with a serious risk of bias [10] was an outlier, and its conclusions were treated with appropriate caution. Our findings are consistent with the broader literature, which has progressively moved away from a dichotomous "for or against" PBD stance towards a more personalized, patient-centric algorithm. For instance, a recent guideline from the International Study Group of Pancreatic Surgery (ISGPS) [30] recommends against routine PBD but endorses it for patients with severe jaundice, cholangitis, or an anticipated delay to surgery, a position that is strongly corroborated by the evidence synthesized in this review.

Limitations

This systematic review has several limitations that must be acknowledged. First, the exclusive inclusion of retrospective cohort studies, despite their robust methodologies in some cases, inherently carries a risk of residual confounding. Unmeasured variables, such as surgeon volume, specific institutional protocols, and nuanced patient comorbidities, could influence outcomes. Second, there was significant heterogeneity in the techniques of PBD (ERCP vs. PTBD), the specific stent types used (plastic vs. metal), and the definitions of outcomes like POPF and DGE across the studies, which precluded a meta-analysis and complicates direct comparisons. Third, the focus on bilirubin as the primary effect modifier, while clearly important, may overlook other significant factors such as nutritional status, patient age, and the presence of specific comorbidities. Finally, the inclusion of studies from diverse healthcare systems introduces potential variability in perioperative care standards that could affect outcome rates.

## Conclusions

The impact of PBD on outcomes after PD is complex and context-dependent. PBD is unequivocally associated with an increased risk of infectious complications, a trade-off that appears unjustified in patients with low or moderate bilirubin levels. However, in the context of severe hyperbilirubinemia, PBD serves as a crucial risk-mitigation strategy, potentially reducing overall morbidity and mortality by alleviating the systemic toxicity of profound jaundice. The timing of drainage further modulates risk, with shorter intervals likely reducing infectious sequelae. Therefore, the decision to employ PBD should not be routine but should be individualized, guided primarily by the patient's serum bilirubin level and clinical condition. Future research should focus on prospectively validating specific bilirubin thresholds, optimizing drainage techniques and timing to minimize infectious risks, and developing integrated protocols that combine judicious PBD with enhanced recovery after surgery pathways.

## Appendices

**Table 4:** Search Strategy Used for Each Database

Database	Search Strategy
PubMed	("Preoperative Care"[Mesh] OR pre-operative[tiab] OR preoperative[tiab] OR preoperativ*[tiab]) AND ("Biliary Tract Surgical Procedures"[Mesh] OR "Cholestasis/surgery"[Mesh] OR biliary drainage[tiab] OR biliary stent*[tiab] OR biliary decompression[tiab] OR ERCP[tiab] OR PTBD[tiab] OR PTCD[tiab]) AND ("Pancreaticoduodenectomy"[Mesh] OR pancreaticoduodenectomy[tiab] OR pancreatoduodenectomy[tiab] OR Whipple procedure[tiab] OR Whipple's procedure[tiab] OR duodenopancreatectomy[tiab]) AND (2019/12/31:2025/12/31[pdat])
Scopus	( TITLE-ABS-KEY ( pre-operative OR preoperative OR preoperativ* ) AND TITLE-ABS-KEY ( "biliary drainage" OR "biliary stent*" OR "biliary decompression" OR ercp OR ptbd OR ptcd ) AND TITLE-ABS-KEY ( pancreaticoduodenectomy OR pancreatoduodenectomy OR "whipple procedure" OR "whipple's procedure" OR duodenopancreatectomy ) ) AND PUBYEAR > 2019 AND ( LIMIT-TO ( DOCTYPE , "ar" ) )
Web of Science	TS=((pre-operative OR preoperative OR preoperativ*) AND ("biliary drainage" OR "biliary stent*" OR "biliary decompression" OR ERCP OR PTBD OR PTCD) AND (pancreaticoduodenectomy OR pancreatoduodenectomy OR "whipple procedure" OR "whipple's procedure" OR duodenopancreatectomy)) Refined by: (Filters for Publication Years: 2020-2025) AND Document Types: (ARTICLE)
Embase	('preoperative period'/exp OR 'pre-operative':ti,ab,kw OR 'preoperative':ti,ab,kw OR preoperativ*:ti,ab,kw) AND ('biliary tract drainage'/exp OR 'biliary stent'/exp OR 'biliary drainage':ti,ab,kw OR 'biliary stent*':ti,ab,kw OR 'biliary decompression':ti,ab,kw OR 'ercp':ti,ab,kw OR 'ptbd':ti,ab,kw OR 'ptcd':ti,ab,kw) AND ('pancreaticoduodenectomy'/exp OR 'pancreaticoduodenectomy':ti,ab,kw OR 'pancreatoduodenectomy':ti,ab,kw OR 'whipple procedure':ti,ab,kw OR 'whipples procedure':ti,ab,kw OR 'duodenopancreatectomy':ti,ab,kw) AND (2020-2025)/py
CINAHL	( (MH "Preoperative Care") OR TI ( pre-operative OR preoperative ) OR AB ( pre-operative OR preoperative ) ) AND ( (MH "Biliary Tract Diseases/SU") OR TI ( "biliary drainage" OR "biliary stent*" OR "biliary decompression" OR ERCP OR PTBD OR PTCD ) OR AB ( "biliary drainage" OR "biliary stent*" OR "biliary decompression" OR ERCP OR PTBD OR PTCD ) ) AND ( (MH "Pancreaticoduodenectomy") OR TI ( pancreaticoduodenectomy OR pancreatoduodenectomy OR "Whipple procedure" OR "Whipple's procedure" ) OR AB ( pancreaticoduodenectomy OR pancreatoduodenectomy OR "Whipple procedure" OR "Whipple's procedure" ) ) AND Published Date: 20200101-20251231
